# Ionogel-based flexible electronics

**DOI:** 10.1093/nsr/nwaf541

**Published:** 2025-12-03

**Authors:** Qinbo Liu, Xu Ou, Yingjie Zhou, Feng Yan

**Affiliations:** State Key Laboratory of Advanced Fiber Materials, College of Materials Science and Engineering, Donghua University, Shanghai 201620, China; State Key Laboratory of Advanced Fiber Materials, College of Materials Science and Engineering, Donghua University, Shanghai 201620, China; State Key Laboratory of Advanced Fiber Materials, College of Materials Science and Engineering, Donghua University, Shanghai 201620, China; State Key Laboratory of Advanced Fiber Materials, College of Materials Science and Engineering, Donghua University, Shanghai 201620, China; Jiangsu Engineering Laboratory of Novel Functional Polymeric Materials, College of Chemistry, Chemical Engineering and Materials Science, Soochow University, Suzhou 215123, China

**Keywords:** ionogel, flexible electronics, sensors, energy harvest, energy storage, smart devices

## Abstract

With the advent of the Internet of Things generation, functional electronics have rapidly emerged as a focus in materials science and electronic engineering. Ionogels have gained significant attention due to their unique physicochemical properties—such as non-volatility, excellent thermal and electrochemical stability, mechanical properties and ionic conductivity—making them crucial materials in flexible electronics. This work focuses on reviewing research progress in the design of ionogels with diverse functionalities and their applications in flexible electronics (primarily over the past five years), compared with previously published reviews. In this review, we comprehensively introduce the fundamental composition and structure of ionogels based on their structure-property-application relationships. Further discussion highlights innovative applications of ionogels in sensors, energy-harvesting devices, storage devices and smart devices, emphasizing their broad potential in flexible electronics. Finally, we propose future directions for ionogels, including multifunctional integration, long-term stability and self-healing capabilities, intended to provide guidance and insights for researchers in flexible electronics.

## Teaser

This review summarizes recent advances in the design and versatile applications of ionogels in flexible electronics, exploring their structure-property relationships and future research directions.

## INTRODUCTION

With the rapid development of the Internet of Things generation, the popularity of smart devices in both daily life and industrial applications imposes higher requirements for electronic materials [1,2]. These materials must exhibit high performance and miniaturization properties and possess flexibility and wearable properties to adapt to evolving usage scenarios and functional requirements [3,4]. Traditional electronic materials, such as silicon, metals and ceramics, although exhibiting excellent electronic performance, are hindered by their rigidity and brittleness, thus limiting the development of flexible-electronics devices (flexible-electronics components or systems that integrate electronic information sensing, conversion, storage or response functions) [5,6]. Alternative novel materials like organic conductors, polymers and liquid metals are developed to achieve a wider range of applications by improving their mechanical flexibility and reducing weight [7]. However, these emerging materials still encounter significant challenges in terms of electrical properties, environmental stability and long-term durability, posing obstacles to their large-scale production and practical applications [8].

**Figure 1.**
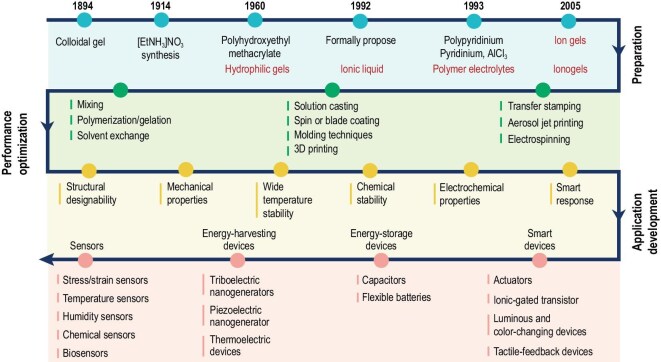
Partial development history of ionogels.

The ionogel consists of the ionic liquid dispersed within a three-dimensional (3D) crosslinked polymer network, which is an innovative soft material [9,10]. In 1894, the emergence of colloidal gels established the foundation for the ‘polymer-network-dispersion-medium’ composite system [11]. Subsequently, the first synthesis of ionic liquids in 1914 (formally named in 1992) provided the core component prototype for ionic conductors [12]. Ionogel was formally proposed in 2005, integrating ‘highly conductive ionic liquids’ with ‘stable 3D polymer networks’ for the first time, thus overcoming the limitations of traditional gels relying on small-molecule solvents and ionic liquids lacking structural support [13]. Subsequent innovations in preparation processes include physical and chemical crosslinking, 3D printing and *in situ* polymerization. By optimizing ionic-liquid types, network architectures (e.g. single, double and semi-interpenetrating networks), and incorporating nanofillers (e.g. MXene, MOF), ionogels have achieved remarkable enhancements in mechanical toughness, ionic conductivity and environmental stability [14]. These properties enable effective ionic transportation pathways while offering excellent thermal stability and chemical resistance. Consequently, ionogels can be widely applied in electrochemical devices and wearable electronics, including flexible sensors, energy-storage devices and smart actuators, serving as a pivotal bridge between material innovation and the practical implementation of flexible electronics (Fig. 1) [9,15]. Thorough research on the composition, performance-enhancement techniques and practical applications of ionogel is expected to continuously promote the advancement of flexible-electronics technology.

**Figure 2.**
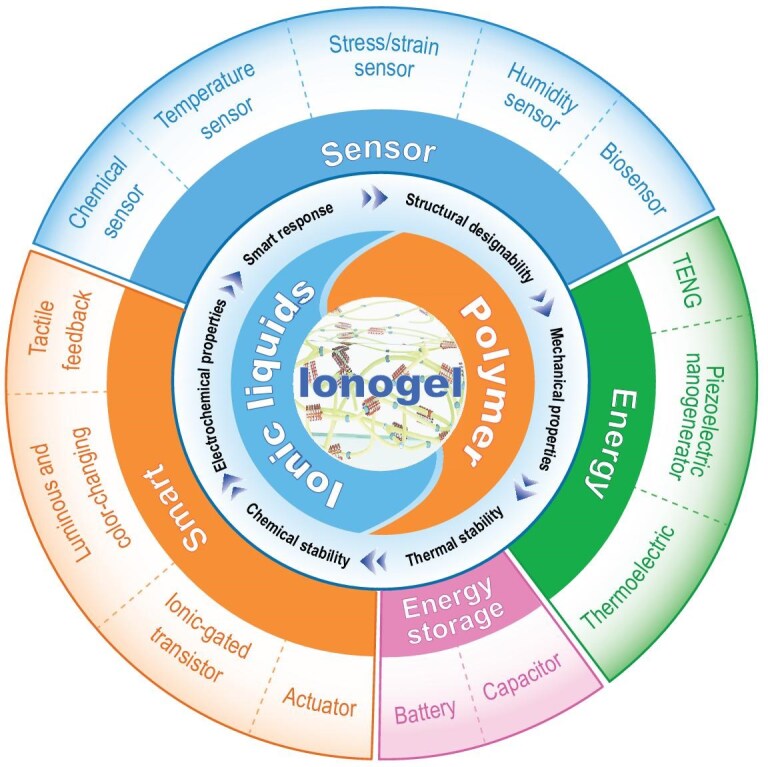
Schematic illustration of this review: the composition and structure of ionogels along with their properties, as well as their applications in different flexible electronics.

With the continuous advancement of digitalization and the increasing emphasis on personal health and lifestyle in recent years, flexible electronics have witnessed rapid development. Among the numerous emerging materials, ionogels have gradually been a focus of research in this field for their unique performance advantages. The research of ionogels in flexible-electronics devices has continued to rise over the past period, and related findings have been emerging continuously. These popular research themes also indicate that ionogels are expected to play an important role in promoting the innovative development of flexible-electronics devices, which prompts us to conduct an in-depth discussion on their specific applications in this field. Several reviews address certain specific properties of ionogels, such as smart response, mechanical properties and electrochemical properties, and discuss in depth the concepts and methods for the design of ionogel enhancements [16–18]. In addition, some reviews focus on the applications of ionogels in some specific areas, such as sensors, energy-storage batteries, ionic-gated transistors and biomedicine [19]. However, to the best of our knowledge, most reviews do not systematically summarize the inter-correspondence between the composition and properties of ionogels for various flexible devices, which is crucial for the design and preparation of ionogels and devices, thus leading to a lack of systematic and comprehensive understanding of different classes of flexible devices. In contrast to previous reviews, as shown in Fig. 2, this work focuses on reviewing research advances in the design of ionogels with different functionalities and their applications in flexible electronics. In this work, we comprehensively introduce the basic composition and structure of ionogels from the structure-property-application interrelationship of ionogels, and further discuss the multiple excellent properties and design methods of ionogels for fully utilizing their advantages in flexible electronics. By comprehensively summarizing the specific applications of ionogels in flexible electronics, we hope to provide an overview for designing ionogels with desired properties and to offer insights that will promote the practical applications of ionogels in flexible electronics.

## FUNDAMENTALS OF IONOGELS

Ionogels are composed of ionic liquids and a polymer network, each playing a crucial role in determining their overall performance. The ionic liquids are primarily responsible for providing excellent electrical conductivity, with their composition and content significantly affecting ion mobility, conductivity and stability. The structures and charge distribution of ionic liquids, along with the combination of anions and cations, can greatly influence the ionic conductivity of the ionogels. Furthermore, the chemical structure of ionic liquids is pivotal in determining the stability of both the ionogels and the ionic liquids, because stable ionic liquids can enhance the thermal, chemical and electrochemical stability [10,20]. The polymer network, in turn, forms a network structure that can be a physical, crosslinked or composite material. This network not only offers mechanical support and shape stability but also interacts with the ionic liquid to influence the performance of ionogels. The selection of ionic liquid and the ionogel network are closely interrelated and together contribute to the overall performance of the ionogel [21,22]. Different network structures—such as linear, branched or crosslinked—along with crosslinking patterns (chemical or physical) and composite morphology, significantly impact the mechanical properties and stability of the ionogel. These factors also affect the combining ability of the polymer framework with the ionic liquid and the ionic transportation efficiency [23,24]. Consequently, the synergy between the ionic liquid and the ionogel network structure shapes the comprehensive performance of ionogels, including their ionic conductivity, mechanical properties and stability, thus enabling them to meet diverse application requirements.

### Ionic liquid in ionogel

**Figure 3.**
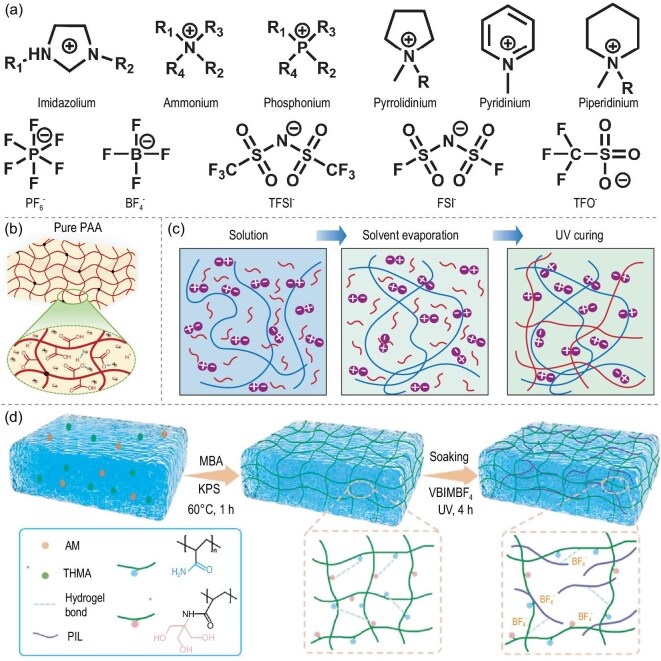
(a) Commonly used cations and anions in ionogels. (b) Poly(acrylic acid) (PAA) swelled in 1-ethyl-3-methylimidazolium ethyl sulfate to form a uniform, elastic PAA ionogel [37]. Copyright 2022, Springer Nature. (c) Schematic illustration of the chemical structure for the double network ionogel [22]. Copyright 2024, Springer Nature. (d) Preparation procedure and multiple interactions within the interpenetrating network structure ionogel [40]. Copyright 2024, Wiley-VCH GmbH.

The selection of ionic liquids in ionogels is closely linked to their performance. Ionic liquids with various chemical structures and properties can influence the performance of ionogels when interacting with the polymer network in many ways. Figure 3a shows the anion and cation structures of ionic liquids commonly used in ionogels. Imidazole cations are usually preferred for their good electrical conductivity and thermal stability [25]. Quaternary ammonium cations offer high ionic conductivity and low toxicity, making them suitable for biomedical applications [26]. Pyridinium cations are favored for their good solubility, strong nucleophilicity and coordination abilities, making them widely used in organic synthesis and biomedical applications [27]. Phosphine salt cations are primarily used in organic synthesis, catalytic reactions and ionic liquids [28]. As for anions, hexafluorophosphate (PF$_{6}$$^{-}$) and tetrafluoroborate (BF$_{4}$$^{-}$) are commonly used in electrochemical devices due to their excellent chemical stability and ionic conductivity [29]. Bis(trifluoromethyl sulfonyl)imide (TFSI$^{-}$) is preferred for electrolyte materials because of its excellent electrochemical stability and electrical conductivity [30]. Chloride and bromide anions provide high electrical conductivity but may be corrosive under certain conditions [31]. Rational selection of ionic liquids (ILs) is essential for the performance optimization of ionogels, as carefully crafting the cationic or anionic structures can significantly improve the properties of the ionogel [32,33].

### Ionogel network

Ionogels feature a variety of network structures, which are formed through crosslinking and interweaving between different polymers or materials. The structures of the ionogel network matrix endow the ionogels with a 3D structure and mechanical integrity, significantly impacting their critical properties such as ionic conductivity, flexibility, mechanical strength, thermal stability and self-healing ability [10]. The mechanical properties of ionogels are significantly influenced by their crosslinking structure. An increase in the degree of crosslinking in the ionogel can enhance its ability to withstand greater stress, thereby improving its rigidity and strength. However, this comes at the cost of reduced flexibility. Conversely, a low degree of crosslinking favors flexibility and tensile strength, although it results in relatively weaker mechanical strength [34,35]. Ionic conductivity in polymer networks is significantly influenced by several factors, including porosity, pore size and network structures. A high degree of porosity, moderate pore size and a well-connected network can enhance the transportation paths, thereby improving conductivity. Additionally, the interactions between the polymer network and the ionic liquid—such as hydrogen bonding between functional groups and ions, and ion-dipole interactions—also contribute to the overall ionic conductivity [24,36].

The network structure of ionogels represents a critical determinant of their unique properties. Ionogels typically exhibit three types of network structures: single-network structures, double-network structures and semi-interpenetrating network structures. The single-network structure represents the fundamental form of ionogel networks, consisting of a continuous 3D network of a single polymer that has been physically, chemically or multiply crosslinked. This structure lacks independent secondary networks, and its properties are solely achieved by regulating the crosslinking characteristics of the single network, which has a limited scope (Fig. 3b) [37]. The double-network structure is an interpenetrating polymer network in which both primary and secondary networks are crosslinked. It is composed of two chemically or structurally distinct crosslinked polymer networks interwoven together (Fig. 3c). This structure allows for the combination of advantages from different network types (such as strength from rigid networks and toughness from flexible networks), enabling synergistic performance enhancement [22,35,38]. Semi-interpenetrating network structures are those in which the primary network is crosslinked while the secondary network remains uncrosslinked. The core characteristic is the coexistence of a ‘primarily crosslinked network’ and an ‘uncrosslinked secondary network’. While the primary network is physically or chemically crosslinked to provide a 3D framework, the secondary network (usually linear or lightly crosslinked polymers) permeates its pores without creating independent crosslinked structures. Both networks are bound together through intermolecular forces, such as hydrogen bonds and ion-dipole interactions (Fig. 3d) [14,39,40]. Each of these different network structures imparts distinct performance qualities to ionogels, and the appropriate network structure and crosslinking type should be reasonably selected based on the specific requirements to the intended application.

The construction of the aforementioned three network structures relies on specific crosslinking methods, and the choice of crosslinking method further determines the mechanical strength, reversibility and environmental stability of the ionogel. Currently, mainstream crosslinking methods can be categorized into three types: physical crosslinking, chemical crosslinking and multi-crosslinking. Physically crosslinked ionogels create stable 3D networks through noncovalent interactions (e.g. hydrogen bonding, electrostatic interactions, van der Waals forces, etc.) [34,41]. These ionogels typically exhibit excellent flexibility, reversibility and recyclability properties. Yan *et al.* [42] developed a physically crosslinked recyclable ionogel by utilizing the porosity and the coordination of metal sites of a metal-organic framework (MOF). The ionogel exhibited a modulus of elasticity and toughness of 58 MPa and 25 MJ m$^{-3}$, respectively. The obtained ionogels can endure an 11 000% stretch, and the energy of rupture was as high as 125 kJ m$^{-2}$, superior to most of the reported ionogels. In addition, they developed high-strength ionogels by leveraging the synergy between force-induced crystallization and halometallate ionic liquids, which formed supramolecular ionic networks. The prepared poly(vinyl alcohol)/halometalate ionic-liquid ionogels exhibited excellent mechanical properties, including ultimate fracture stress ($63.1\pm 2.1$ MPa), strain ($5248\%\pm 113\%$) and unprecedented toughness ($1947\pm 52$ MJ m$^{-3}$), which were much higher than those of most metals and alloys [43]. Physically crosslinked ionogels exhibit remarkable reversibility, achieving restoration of mechanical and functional properties through dynamic cleavage and reformation of noncovalent bonds, while still meeting device operation thresholds. This makes them promising candidates for diverse applications in smart materials [34,44]. Despite their ease of preparation, modifiability and reversible nature, the relatively weak mechanical strength and stability of ionogels restrict their broader application.

The polymer chains in chemically crosslinking ionogels are generally linked by covalent bonds, creating a stronger and more stable 3D network. Chemical crosslinking enhances mechanical strength and thermal stability but is typically irreversible once established [45]. Yan *et al.* [46] constructed a tough and impact-resistant poly(ionic liquid) (PIL)/poly(hydroxyethyl acrylate)/poly(hydroxyethyl acrylate) double-network elastomer through multiple crosslinking of polymer networks and cation-$\pi$ interactions of PIL chains. Benefiting from the strong noncovalent cohesion achieved by the cation-$\pi$ interactions in the PIL chains, the prepared PIL DN elastomers exhibited remarkable compressive strength ($95.24\pm 2.49$ MPa) and toughness ($55.98\pm 0.66$ MJ m$^{-3}$) under a high-speed impact load (5000 s$^{-1}$). Moreover, the synthesized PIL DN elastomers with high strength and excellent flexibility can protect fragile items from impact. Chemically crosslinked ionogels exhibit excellent thermal stability as well as tunable physicochemical properties, making them promising for a wide range of applications, including energy storage (batteries and supercapacitors), sensors and drug-delivery systems [21,47]. However, the limitations of this crosslinking approach should not be neglected, as later modification of the ionogel results in reduced flexibility compared with the desired performance.

The multi-crosslinked ionogel incorporates the advantages of physical and chemical crosslinking, integrating stable covalent bonds with flexible noncovalent interactions. This multi-crosslinked approach ensures that the ionogels retain a certain level of mechanical strength while preserving their flexibility and adaptability [14,22,48]. Consequently, these ionogels exhibit a balance between structural integrity and functional versatility. Liu *et al.* [49] utilized the block copolymer polystyrene-poly(ethylene oxide)-poly(styrene) (PS-PEO-PS) self-assembled in mixed ionic liquids of [1-(4-vinyl benzyl)-3-butylimidazoliumc bis(trifluoromethyl sulfonyl)imide ([VBBIm]NTf$_{2}$) and 1-ethyl-3-methylimidazolium bis(trifluoromethyl sulfonyl) imide ([EMIm]NTf$_{2}$)] to obtain a physically crosslinked network. This network was further processed using methods such as inkjet printing, spraying and 3D printing to prepare the physically-chemically crosslinked ionogels with complex structures. The resulting ionogels exhibit superior mechanical properties compared with those of the single physically or chemically crosslinked ionogels, with a tensile strength of 0.29 MPa at a strain of over 400% and a compressive strength of 10 MPa at 85% strain. The multi-crosslinked ionogel combines strength stability and reversible adjustability, effectively enhancing the flexibility, stability and electrical performance of flexible electronics and enabling better adaptation to a variety of complex application scenarios.

## PROPERTIES OF IONOGELS

**Figure 4.**
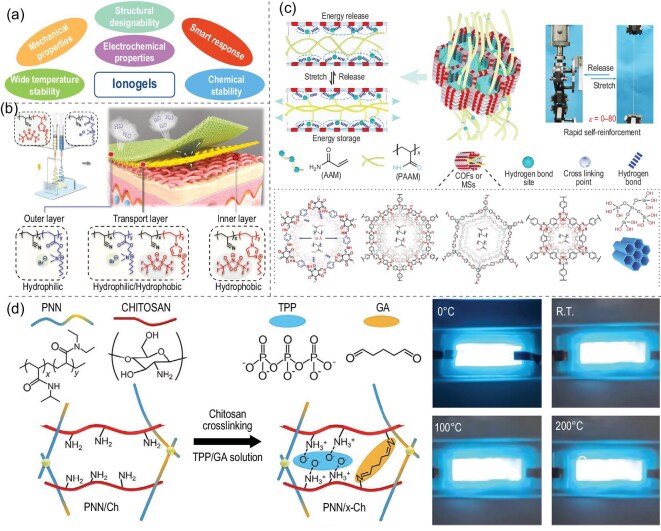
(a) Representative merits of ionogels. (b) The fabrication and application of the PIL membrane electronic skin with moisture-wicking, breathable and antibacterial qualities for bioelectrical signal monitoring [52]. Copyright 2021, Wiley-VCH GmbH. (c) Schematic illustration of the hysteresis-free and crack-propagation-insensitive hydrogels, ionogels and organogels prepared by the nanoconfined polymerization strategy [56]. Copyright 2023, Springer Nature. (d) Illustration of the network structures for poly(N-isopropylacrylamide-co-N, N’-diethylacrylamide) (PNN)/chitosan and PNN/crosslinked chitosan, and the photographs of light-emitting alternating-current electroluminescent devices operated at various temperatures [35]. Copyright 2021, Wiley-VCH GmbH.

In comparison to conventional gels, ionogels exhibit superior thermal stability and chemical resistance, as well as outstanding performance in ionic conductivity, mechanical strength and toughness, enabling their exceptional application in flexible-electronics devices and high-performance protective materials. The performance of ionogels depends on the synergistic effect of ILs and the network matrix, which is influenced by their interactions and micro/macrostructure. Because of these unique advantages, ionogels demonstrate excellent performance in flexible electronics. The outstanding designability of their structures permits precise control over various properties. By carefully constructing different network structures—including single, double or semi-interpenetrating networks—they can meet the diversified requirements of flexible electronics. Regarding mechanical properties, ionogels provide good flexibility and stretchability, which can adapt freely to the deformations of flexible-electronics devices, offering strong support for their stability. In complex environments where there might be contact with sweat, chemical reagents and other scenarios, their performance can be maintained, ensuring that flexible-electronics devices are not adversely affected. Meanwhile, the excellent electrochemical properties of ionogels provide efficient channels for electrochemical processes, such as electrical energy transmission and signal conduction within flexible-electronics devices, thereby ensuring their proper functioning. In addition, ionogels exhibit stimulus-responsive properties and can sensitively respond to specific external stimuli, such as temperature or pressure. This capability can be innovatively applied in flexible sensors and other devices to enable accurate monitoring of environmental changes. As shown in Fig. 4a, all these outstanding properties allow ionogels to play an indispensable role in numerous application scenarios within the realm of flexible electronics, making them an essential material for advancing flexible-electronics technology.

### Structural designability

The designability of the structure sets ionogels apart from traditional materials and offers a broad space for performance tuning. Through molecular design, their physicochemical properties can be customized by adjusting the cations and anions of ionic liquids, including ion mobility, viscosity, electrochemical stability and thermal stability [14]. For example, modifying the alkyl chain length of the cation or introducing functional groups could effectively modulate the viscosity and electrical conductivity of the ionic liquid, which in turn influences the overall performance of the ionogel [50,51]. The hydrophilic and hydrophobic properties of the material can be tuned by adjusting the anion types (Fig. 4b) [52]. In addition, the pore size, mechanical strength, flexibility and functionality of the ionogel network can be modified by adjusting the ratio, distribution, crosslink density and network structure of the nanomaterials and polymer. Niu *et al.* [53] developed a multifunctional ionogel (API) enhanced by IL-modified zinc oxide (IMZnO). The IMZnO effectively promoted the internal consumption among molecules due to its modification-boosted affinity and dispersibility, leading to a stronger interfacial bond and enhanced conductivity. The performance of the API can be regulated by the IL content. A high IL content resulted in excellent flexibility (1418%) and conductivity (0.31 S m$^{-1}$), endowing the material with excellent strain-sensing properties. The API with a lower IL content exhibits noteworthy shape-memory properties, providing thermal responsiveness that is suitable for applications in temperature-alarm devices. The versatility and tunability of PILs further enhance the capacity to customize the ionogel properties, making them applicable in diverse areas such as high-performance batteries, electrolyte materials, flexible sensors and other fields [40]. By rationally designing the structures of ionic liquids and ionogel networks, the electrical conductivity, mechanical properties, thermal stability and electrochemical properties of ionogels can be optimized to meet different application requirements.

### Mechanical properties

The mechanical properties of ionogels are determined primarily from the ionogel network structure, and the flexibility, strength and elasticity can be regulated through factors such as polymer type, crosslinking density, additives and network structure [54,55]. The excellent flexibility of ionogels enables them to withstand deformations such as bending, folding and stretching, making them ideal candidates for flexible-electronics devices. As illustrated in Fig. 4c, Yan *et al.* [56] proposed a nanoconfined polymerization strategy based on strong hydrogen-bonding interactions between closed nano-channels and polymer fragments. This approach effectively immobilizes polymer chain segments that might slide under loading by introducing covalent organic frameworks or molecular sieves (MS) with interpenetrating polymer chain segments, thereby avoiding energy dissipation, alleviating transient stress concentration at cracks and preventing crack extension. Using this strategy, highly flexible ionogels with ultimate fracture strain ($17\, 580\%\pm 308\%$), toughness ($87.7\pm 2.3$ MJ m$^{-3}$) and crack extension strain (5800%) were achieved.

The introduction of nanomaterials (graphene, carbon nanotubes or nanoparticles) into the polymer matrix of ionogels can significantly improve their mechanical properties [57,58]. Inspired by the mechanical reinforcement of natural biomacromolecules through noncovalent aggregates, Yao *et al.* [59] proposed a strategy to construct nanofibril-based ionogels through complex coacervation-induced assembly. Cellulose nanofibrils (CNFs) can bundle together with PIL to form a superstrong nanofibrous network, in which the ionic liquid (CNF-PIL-IL) is retained, resulting in ionogels with high liquid inclusion and ionic conductivity. The strength of CNF-PIL-IL ionogels can be tuned by varying the IL content, reaching values of up to 78 MPa. These nanomaterial-enhanced ionogel networks show potential for diverse applications in biomedicine, flexible electronics and environmental governance due to their improved mechanical properties and functionality.

### Wide temperature stability

Ionogels demonstrate exceptional and stable performance across a broad temperature spectrum, from low to high temperatures, due to their unique thermal properties and the synergistic interactions between their components. The ionic liquids within ionogels possess distinctive thermal characteristics, such as a low freezing point and a wide liquid range, which contribute significantly to their electrical, mechanical and microstructural properties. Furthermore, the interactions between the ionic liquids and the polymer network, including hydrogen bonding and electrostatic attraction, enhance these properties, ensuring that the ionogels sustain their performance across diverse thermal conditions [60]. Through the selection of suitable ionic liquids and polymer networks, ionogels with a wide temperature applicability range can potentially be prepared for sensors, batteries and supercapacitors in high-temperature environments [61,62]. As shown in Fig. 4d, Yoon *et al.* [35] prepared a conductive ionogel containing a double network of poly(N-isopropylacrylamide-co-N, N’-diethylacrylamide)/chitosan based on covalent crosslinking and ionic crosslinking in highly stable ionic liquids. The assembled electroluminescent devices maintained stable luminescence performance even after 1000 stretch-release cycles or at temperatures up to $200\, ^\circ$C.

On the other hand, the stability of ionogels at a lower temperature can be attributed to their unique composition and internal interaction mechanisms. The ionic liquids exhibit low freezing points due to the nature of ionic bonds and the specific electrostatic interactions between anions and cations. These properties ensure that the ionogel maintains a quasi-solid state and stable ion-transport performance at low temperatures, thereby preventing phase transitions and ion-conduction blockages caused by temperature fluctuations. In addition, interactions between the polymer network with the ionic liquid (e.g. hydrogen bonding and electrostatic attraction) provide not only structural support but also help lower the freezing point of the ionogel, maintaining stability under low-temperature conditions [63,64]. Jiang *et al.* [65] developed high-performance ionogels through the strategic design of water-induced competitive hydration interactions involving 1-butyl-3-methylimidazolium chloride, water and cellulose nanofibers. By carefully balancing the water content within the ionogel, its ionic conductivity could be enhanced while the interactions between cellulose nanofibers and ionic liquids were decreased. This balance also suppresses the freezing and evaporation of water, thereby enhancing the environmental stability of the ionogels. As a result, the prepared ionogels exhibited remarkable performance in extreme conditions, retaining their functionality over a wide temperature range from $-45\, ^\circ$C to $75\, ^\circ$C in low-humidity environments (RH $<15\%$). These properties underscore the excellent anti-freezing and anti-drying capabilities of the ionogels.

### Chemical stability

**Figure 5.**
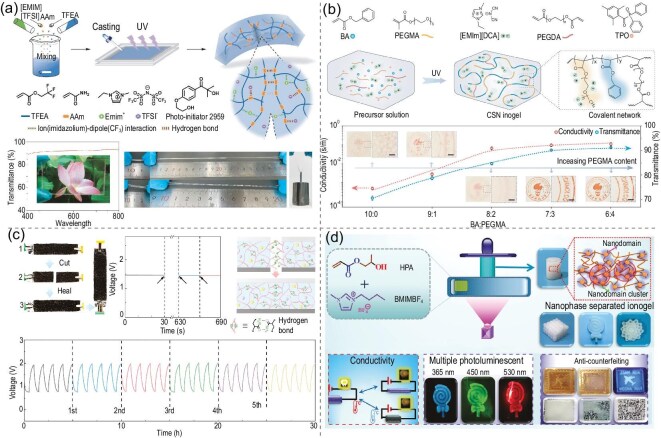
(a) Schematic preparation of ionogels via one-pot, photoinitiated polymerization of 2,2,2-trifluoroethyl acrylate and acrylamide in a hydrophobic ionic liquid, and their transparency and mechanical properties [41]. Copyright 2021, Wiley-VCH GmbH. (b) Illustrations of the components and photopolymerization process of the highly conductive, stretchable, nanostructured ionogels, and comparison of the conductivity and transmittance of ionogels with different poly(ethylene glycol) methyl ether methacrylate contents [24]. Copyright 2024, Springer Nature. (c) Photographs, open-circuit voltage curves, schematic of the self-healing mechanism and galvanostatic charge/discharge curves of the self-healing and wide-temperature flexible ZABs [74]. Copyright 2024, Wiley-VCH GmbH. (d) Chemical structures and a schematic diagram of 3D-printed, nanophase-separated ionogels, and digital photographs of the ionogels illustrating their high stretchability, ultra-low hysteresis, good conductive-strain/temperature responsiveness, unique thermochromic behaviour and multiple photoluminescent properties [78]. Copyright 2024, Wiley-VCH GmbH.

The chemical stability of ionogels is dependent on the chemical inertness of the ionic liquids and polymer networks. With good chemical inertness, ionic liquids and many polymer networks are resistant to a wide range of chemical environments, including acids, bases and solvents [66]. Yue *et al.* [41] prepared a multifunctional ionogel with solvent resistance, recyclability, high electrical conductivity, underwater self-healing ability and underwater adhesion via one-step photoinitiated polymerization of 2,2,2-trifluoroethyl acrylate (TFEA) and acrylamide (AAm) in the hydrophobic ionic liquid [Emim][TFSI] (Fig. 5a). The highly solvent-resistant nature of the ionogel allows it to preserve its structure and properties when exposed to diverse organic solvents and water, which is crucial for application in chemical sensors and electrochemical batteries [9,21].

### Electrochemical properties

The electrochemical performance of ionogels is crucial for their application in energy storage and conversion devices, particularly in terms of electrical conductivity and electrochemical stability [67,68]. The typical conductivity range of ionogels under ambient conditions is 10$^{-5}$ to 10$^{-2}$ S cm$^{-1}$, depending on carrier concentration and mobility. This is significantly influenced by multiple intrinsic material properties, including ion-source characteristics (ionic-liquid type and concentration), polymer-matrix structure and performance, nanoparticle fillers or additives, and external environmental factors. Ionic liquids typically exhibit high ionic mobility, benefiting from their unique ionic structure and weak interaction forces [69–71]. As shown in Fig. 5b, Ge *et al.* [24] proposed a UV-curable ionogel with a bicontinuous nanostructure, capable of achieving high conductivity without sacrificing printability and mechanical properties. The prepared CSN ionogel simultaneously exhibits high ionic conductivity (over 3 S m$^{-1}$), high stretchability (over 1500%), low hysteresis (0.4% at 50% strain), thermal stability over a wide temperature range ($-72\, ^\circ$C to $250\, ^\circ$C) and moisture resistance. The higher ionic conductivity of ionogels enables a wide range of promising applications in electrochemical devices, including batteries, supercapacitors, fuel cells, sensors and electrochromic devices [36,41,72].

Meanwhile, the excellent electrochemical stability of ionogels ensures stable and reliable operation of flexible devices during electrochemical processes and extends their lifetime [73]. As shown in Fig. 5c, Li *et al.* [74] synthesized a self-healing ionogel via photopolymerization of acrylamide and poly(ethylene glycol) monomethyl ether acrylate in 1-ethyl-3-methylimidazolium dicyanamide with zinc acetate dihydrate. This ionogel was first used as an electrolyte to fabricate self-healing zinc-air batteries (ZABs), demonstrating an ultra-long cycle life and excellent stability under harsh conditions. In addition, the fast response capability and lower resistance of ionogels enhance the activity of electrochemical interfaces and improve the energy conversion ratio [75]. The electrochemical properties of ionogels can be further optimized through rational design of their composition and structure, enabling broad application prospects in high-performance batteries, supercapacitors and sensors.

### Smart response

By rationally modifying the ionic-liquid structure or polymer framework, ionogels with various stimulus-responsive properties can be obtained. These responsive ionogels, with their tailored material structures and compositions, can quickly and reversibly respond to external stimuli (e.g. temperature, pH, light, electric field and ion concentration), resulting in changes to their physical and chemical properties, including size, shape or conductivity [76,77]. As shown in Fig. 5d, Song *et al.* [78] fabricated non-covalently crosslinked ionogels with high stretchability and ultra-low hysteresis based on phase separation by 3D printing 2-hydroxypropyl acrylate (HPA) in 1-butyl-3-methylimidazolium tetrafluoroborate ([BMIM][BF$_{4}$]). Because of the formation of nanophase-separated and crosslinked structures, the prepared ionogels displayed unique thermochromic and multiple photoluminescent properties, which can be synergistically employed for anti-counterfeiting and encryption. Notably, flexible thermo-mechanical multi-peak vision ion sensors for strain and temperature sensing were fabricated, exhibiting a highly stable and reproducible electrical response over 20 000 cycles and demonstrating synergistic optical and electrical output properties. Such intelligent responsiveness enables ionogels to adaptively regulate their properties during environmental fluctuations, particularly demonstrating potential for extensive applications in biosensors, drug-delivery systems and flexible-electronics devices [79]. Thus, as a newly emerging material, ionogels are gradually becoming an important focus in materials science research.

In conclusion, ionogels occupy a significant position in the field of materials due to their unique, comprehensive performance. The designability of their structures allows customization to meet diverse application requirements, while their mechanical properties enable adaptation to various mechanical environments. Their stability across a wide temperature range and their chemical stability ensure reliable operation under extreme conditions. Additionally, their electrochemical properties make them promising candidates for energy storage and conversion applications, and their responsiveness offers potential for intelligent applications. These properties are synergistic and complementary, collectively forming the robust advantages of ionogels. As scientific research deepens and technological innovations continue, ionogels are anticipated to achieve breakthroughs and broaden their applications in energy, smart materials and extreme environments. This expansion will further unlock their potential as multifunctional materials, providing high-quality and efficient solutions to complex engineering and technological challenges.

## IONOGEL APPLICATIONS IN FLEXIBLE ELECTRONICS

Flexible electronic devices have become a prominent research focus across various fields in the current era of rapid advancements in electronic technology, owing to their unique attributes such as bendability and foldability. Among the materials being explored for these applications, ionogels stand out for their potential in flexible electronics. Ionogels can be effectively utilized in a wide array of applications, offering considerable potential to enhance performance and expand functionality. As shown in Fig. S1, their versatility is particularly evident in areas such as sensing, energy collection, energy storage and intelligent response mechanisms, where they can contribute to the development of more advanced and efficient flexible-electronics devices.

### Sensors

Because of their superior electrical conductivity, mechanical flexibility, biocompatibility and stimulus responsiveness, ionogels exhibit broad application potential in the field of sensors. These materials could be utilized in flexible sensors capable of detecting a variety of physical, chemical and biological signals, including temperature, pressure, strain, humidity, chemicals and biomolecules. By integrating ionogels with different sensing technologies, diverse sensor designs can be achieved.

#### Stress/strain sensors

**Figure 6.**
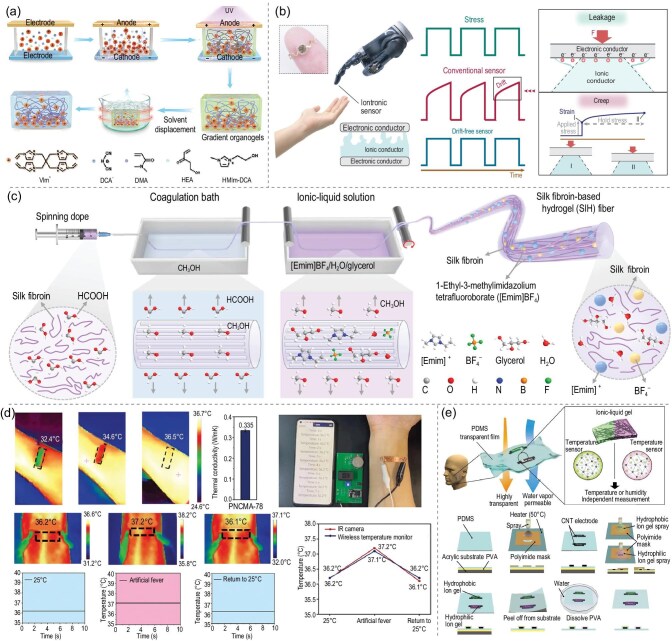
(a) Schematic illustration of the process that employs electric field induction and a solution-displacement strategy to prepare gradient-inspired gradient ionogels [91]. Copyright 2021, Wiley-VCH GmbH. (b) Illustration of ion sensors as artificial skin for human-machine interaction, in which drift-free ion sensors generate non-drifting signals for accurate sensing. Traditional ion sensors generate drifting signals, leading to inaccurate sensing due to square-wave stress, leakage and/or creep of the ionic conductor [92]. Copyright 2024, Springer Nature. (c) Scheme diagram for the fabrication of silk-fibroin-based ionic hydrogel fibers [97]. Copyright 2024, Springer Nature. (d) Wireless temperature monitor based on poly(N-cyanomethyl acrylamide) deep eutectic ionogel, including its thermal conductivity, a digital photo of the temperature monitor, infrared images of captured body-surface temperature, temperature-time curves obtained from mobile-phone data and temperature-change curves [103]. Copyright 2023, Wiley-VCH GmbH. (e) Concept of an ionogel-based thin-film sensor for temperature and humidity and its fabrication process [107]. Copyright 2022, Wiley-VCH GmbH.

The ionogel plays an important role in stress/strain sensors. When subjected to pressure, the internal ionic transportation channels of ionogels are altered, leading to changes in electrical parameters such as resistance and capacitance [80]. This property enables ionogels to detect small pressures with high sensitivity, making them suitable for monitoring weak physiological signals in the human body [81–83]. By designing structures and compositions tailored to different pressure ranges, such as multi-phase ionogels, they can maintain effective response characteristics across a wide spectrum [84,85]. Additionally, their high flexibility and wearability allow them to be integrated with flexible substrates, enabling sensors to adhere to irregular surfaces like human skin [86–88]. This capability of ionogels facilitates the real-time monitoring of human movements and positions in wearable electronics [89,90]. Yan *et al.* [91] prepared gradient ionogels by injecting a precursor solution containing a tetravinyl cationic crosslinker between the cathode and the anode. They utilized an adjustable power supply to maintain a stable electric field and employed a printable strategy that was activated by an electric field with a concentration gradient (Fig. 6a). The gradient concentration of the cationic crosslinker resulted in a modulus gradient in the ionogels. These flexible ionoelectronic sensors based on the gradient ionogels, demonstrated high sensitivity, a broad detection range (from 300 Pa to 2.5 MPa) and excellent reliability. This variation can be used as an indicator of stress or strain, enabling the accurate monitoring of material deformations. Guo *et al.* [92] rationally designed and synthesized a polyelectrolyte elastomer that is free from leakage and creep at the molecular level through cationic grafting and copolymerization with neutral sliding-chain segments, thereby enabling drift-free ion sensing. Plasmonic sensors constructed from this polyelectrolyte elastomer exhibited an initial drift rate of 0.01–0.1% min$^{-1}$ at 500 kPa, which decreased to 0.001% min$^{-1}$ within 10 min. This performance is two to four orders of magnitude better than that of sensors using other ionic conductors (Fig. 6b). This property enables ionogels to be widely used in wearable devices and structural health monitoring, offering innovative solutions to enhance the safety and reliability of these devices.

Furthermore, the flexibility, stretchability and multifunctionality of ionogels make them an ideal choice for smart textiles. By incorporating ionogels into fabrics, textiles can be equipped with smart features such as sensing, energy harvesting, energy storage, light emission, color change and self-healing [93]. These properties extend the applications of conventional textiles and open up new opportunities in fields such as wearable technology, healthcare and human-computer interaction [94–96]. As shown in Fig. 6c, Zhang *et al.* [97] reported a smart textile based on conductive silk-fibroin ionogel fibers. Benefitting from the internally oriented structure of the fibers and ionic bonding, the textile possessed superior fiber-breaking strength (55 MPa), ductility (530%), stability and good electrical conductivity (0.45 S m$^{-1}$), enabling it to respond electrically to external hazards, accurately sense human touch, provide real-time monitoring of the user’s status, and, to some extent, offer energy support, thereby greatly enhancing the user experience and the product value.

#### Temperature sensors

Ionogels hold significant promise in the field of temperature sensors due to their excellent temperature sensitivity, where temperature changes affect internal ion migration and phase transitions within the ionogel, resulting in variations in resistance and capacitance [77,98–101]. This property forms the foundation of their temperature-sensing capability. Furthermore, ionogels demonstrate stability across different humidity environments, fulfilling the requirements of flexible-electronics devices that require high-temperature accuracy. Additionally, their flexibility and stretchability make ionogels suitable for producing wearable temperature sensors. This adaptability allows them to conform closely to human skin, ensuring precise monitoring of body temperature during daily activities and sports, thereby providing accurate and reliable temperature measurements [102]. As shown in Fig. 6d, Liu *et al.* [103] prepared polymerizable deep eutectic solvents (DESs) by mixing N-cyanoethyl acrylamide, which contains an amide group and a cyano group on the same side chain, with lithium bis(trifluoromethane) sulfonimide. The prepared DESs were then polymerized to obtain supramolecular deep eutectic ionogels. The supramolecular ionogels exhibited excellent mechanical properties, strong adhesion, environmental stability and high-temperature sensitivity. Moreover, they have been utilized for the development of a wireless-based temperature monitor with an excellent high-temperature coefficient of resistance value (8.4% K$^{-1}$) over a wide temperature detection range. These properties make the ionogels highly effective for real-time temperature monitoring in various industrial and environmental applications. As advancements are made in the optimization of ionogel performance, there is considerable potential for accurately detecting minute temperature fluctuations in demanding sectors. By integrating multiple technologies, the development of multifunctional smart temperature sensors is feasible [104], offering advanced sensing solutions for cutting-edge applications.

#### Humidity sensors

Ionogels are mainly composed of polymer networks and ionic liquids, which are rich in mobile ions. They interact with moisture in the surrounding air at different humidity levels. As water molecules from the air penetrate the ionogel, the ion concentration, ion mobility and other physicochemical properties inside the ionogel are altered, leading to significant changes in its electrical properties, such as resistivity. By accurately measuring these changes in resistivity, it is possible to precisely determine the environmental humidity [90,105,106]. As shown in Fig. 6e, Hiroki *et al.* [107] assembled a transparent, breathable and flexible ionogel thin-film sensor for temperature and humidity, equipped with two sets of carbon nanotube (CNT) transparent electrodes and ionogel on a flexible polydimethylsiloxane thin-film substrate. The sensor can independently detect temperature and humidity, with hydrophobic and hydrophilic ionogels deposited on the two sets of CNT electrodes serving as temperature- and humidity-sensitive layers, respectively. The sensor exhibited sensitivities of 15.4% $^\circ$C$^{-1}$ for temperature and 2.0% per percentage of relative humidity. Humidity-sensing technology based on ionogels holds significant application potential in climate monitoring and agricultural environment regulation, improving responsiveness to environmental changes.

#### Chemical sensors

**Figure 7.**
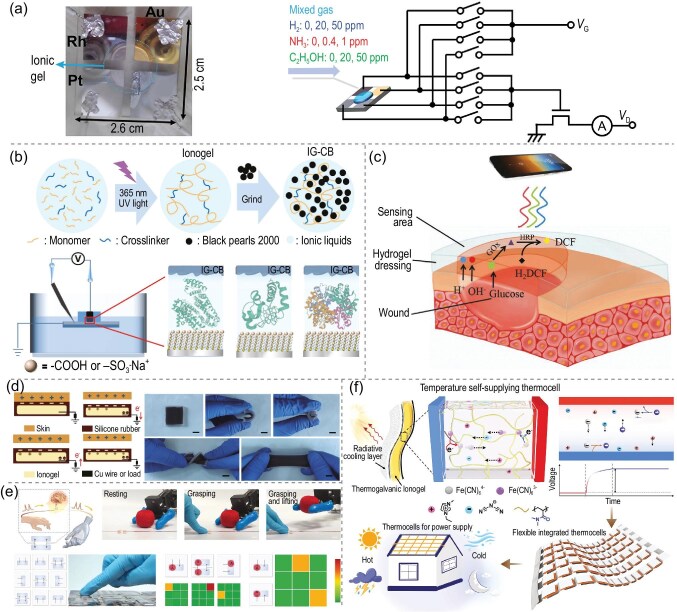
(a) Photograph of the ionogel sensor with multiple electrodes and a schematic of the sensor mechanism [111]. Copyright 2022, American Chemical Society. (b) Schematic illustrations of the fabrication process of ionogel electrodes and the formation of protein junctions [114]. Copyright 2023, Wiley-VCH GmbH. (c) Schematic illustration of a poly-carboxy betaine (PCB) hydrogel dressing for detecting pH and glucose concentration in wound exudate [115]. Copyright 2019, Wiley-VCH GmbH. (d) Working mechanism of the SI-TENG in single-electrode mode, and digital images of SI-TENG under different mechanical deformations [123]. Copyright 2023, Springer Nature. (e) Demonstration of the integration of two parallel piezo-ionic elastomers with a robotic arm in various states, including a schematic of the piezo-ionic array sensing pressure, processing the input signal and exhibiting color changes when different pixels are pressed [130]. Copyright 2024, Wiley-VCH GmbH. (f) Schematic representation of a thermogalvanic ionogel-based thermocell for all-weather power generation [143]. Copyright 2024, Wiley-VCH GmbH.

Ionogels are employed in chemical sensing to detect toxic gases, volatile organic compounds, biomolecules and humidity effectively, owing to their high ionic conductivity. This property allows ionogels to respond rapidly to changes in environmental chemicals. Upon interaction with specific gases or liquids, their internal properties undergo significant alterations, facilitating the sensitive detection of complex chemical environments [108–110]. As shown in Fig. 7a, Uchida *et al.* [111] reported a gas-composition sensor based on [EMIM][BF$_{4}$] ionogels, in which the absorption of gases by the ionogel directly affects the electrode voltages, allowing simultaneous detection of H$_{2}$, NH$_{3}$ and C$_{2}$H$_{5}$OH concentrations from the voltage signals between each set of electrodes in the device. This characteristic enables the use of ionogels in gas sensors and liquid sensors, particularly for environmental monitoring and industrial process control, demonstrating significant application potential. Yu *et al.* [112] designed and synthesized a gelator containing tripyridine and imidazolium salt units, which were integrated into an ionogel in three ionic liquids through a process of heating and cooling. The ionogel containing pH-sensitive dye (bromothymol blue and methyl red) was employed as a colorimetric sensor to monitor the total volatile basic nitrogen (TVB-N) in meat at $-4\, ^\circ$C, allowing straightforward and reliable assessment of meat quality by visual identification. The application of ionogels in chemical sensing holds considerable promise and may drive the continuous development of chemical sensors toward miniaturization, intelligence, multifunctionality and high stability.

#### Biosensors

The specific recognition of target biomolecules can be achieved by introducing biorecognition molecules, such as antibodies or nucleic acid aptamers, into the ionogel. These biorecognition molecules can specifically bind to the target biomolecules, enabling the ionogel sensor to exhibit high selectivity and accurately detect the target biomolecules in complex biological samples without interference from other contaminants [113]. For example, Li *et al.* [114] developed a highly conductive, rubber-like electrode (IG-CB) using an ionogel composed of an acrylic monomer, the ionic liquid ([C$_{2}$mim][EtSO$_{4}$]) and conductive carbon powder (Fig. 7b). IG-CB was employed as a top contact to form highly reproducible protein connections (human serum albumin, cytochrome C and hemoglobin). Structural analysis of proteins adsorbed on self-assembled monolayers using surface spectroscopy and microscopy provided a detailed understanding of the tunneling effect of proteins. Meanwhile, ionogels show great potential in medical rehabilitation. As shown in Fig. 7c, Zhang *et al.* [115] developed a multifunctional amphoteric ionogel for the simultaneous detection of two fluctuating wound parameters, pH and glucose. The ionogel integrated a pH indicator dye (phenol red) and glucose-sensing enzymes (glucose oxidase and horseradish peroxidase) within an amphoteric ionic polycarboxylated betaine. Both glucose-sensing enzymes showed improved activity and stability in artificial wound exudate. In addition, visible RGB images were collected using a smartphone, and fitted equations were obtained to monitor glucose and pH changes in wounds in diabetic rats. The ionogel is anticipated to achieve high sensitivity, specific recognition, multimodal sensing and real-time monitoring in biomolecular sensing, which is anticipated to be highly valuable in the biomedical field.

### Energy-harvesting devices

Nowadays, environmental and energy issues are becoming a global concern as technological development and energy demand continue to increasing. Numerous innovative energy-harvesting devices are emerging to explore more diversified ways of obtaining energy and to improve energy-utilization efficiency for sustainable development. The migration of ions in ionogels can be precisely regulated owing to their high structural tunability. In this context, ionogels have been extensively developed for use in energy-collection devices, leveraging their tunable electrical properties and excellent flexibility in flexible energy-harvesting devices, such as triboelectric nanogenerators, piezoelectric nanogenerators and thermoelectric generators, to effectively capture and recycle energy.

#### Triboelectric nanogenerators

Triboelectric nanogenerators (TENGs) are devices that converts mechanical energy into electrical energy using friction initiation and electrostatic induction effects [116–118]. Ionogels have diverse applications in nanofriction generators, primarily owing to their high electrical conductivity, flexibility and stretchability, which make them ideal as electrode materials. These properties ensure efficient charge transfer and the reliable operation of generators across various scenarios. Additionally, ionogels serve as effective friction-layer materials, capable of generating abundant friction charges while maintaining stability and durability [119]. Furthermore, their ability to integrate with other devices enhances their multifunctionality, making them particularly suitable for constructing self-powered systems in wearable devices, thereby providing essential energy support for their operation [120]. Sun *et al.* [121] synthesized acrylic acid (AA)-vinyl imidazolium dicyandiamide ([Emim][OAc])-zinc acetate-zinc oxide nanoparticles self-healing ionogels via *in situ* polymerization. The self-healing ionogels exhibited high mechanical strength, compressive strength, transparency and favorable ionic conductivity, attributed to the presence of ZnO nanoparticles and their good compatibility with PAA and [Emim][OAc]. The ionogel-based TENG, with a maximum output power density of 3.15 W m$^{-2}$, was obtained by assembling the self-healing ionogel with 3 M adhesive tape, enabling the repair of its output electrical properties following damage. The large number of mobile ions within the ionogel can freely shuttle under an electric field, offering high electrical conductivity. This characteristic ensures efficient charge transfer and collection when used as an electrode material for nanofriction generators, thereby enhancing the output performance. By optimizing the composition, structure and surface morphology of the ionogel, the output performance of the TENGs can be further improved, including enhancements in output voltage, current and energy-conversion efficiency [122]. Sun *et al.* [123] developed a high-temperature stability, stretchability and washability stretchable ionogel-based triboelectric nanogenerator (SI-TENG) using ionogel as the electrode and silicone rubber as the friction and encapsulation layers. As shown in Fig. 7d, the SI-TENG can serve as a self-charging power source to drive electronic calculators or charge commercial capacitors by harvesting biomechanical energy. Because of its unique physicochemical properties, the ionogel effectively improves energy-conversion efficiency, enhances the flexibility and stability of equipment, and provides a novel approach to delivering reliable self-power for wearable and flexible-electronics devices.

#### Piezoelectric nanogenerator

Piezoelectric generators convert mechanical energy into electrical energy by utilizing the piezoelectric effect of piezoelectric materials [124–126]. Ionogels serve not only as matrix materials for piezoelectric materials to enhance their flexibility but also as electrode materials for improving their output performance. The composite piezoelectric structure used in the construction of the ionogel can be optimized by modifying the internal electric field distribution. This modification enhances the piezoelectric effect of the materials, thereby improving the output voltage and current of the generator. Additionally, the inherent toughness and elasticity of the composite structure contribute to improved mechanical properties, enabling it to withstand greater mechanical stresses and deformation. Consequently, these enhancements increase the reliability and stability of the composite structure in complex mechanical environments [127–129]. Wu *et al.* [130] reported a piezoelectric ion damper based on 1-ethyl-3-methylimidazolium bis(trifluoromethylsulfonyl)imide and ionic plastic crystals, which internally form a finely tuned microphase-separation structure with an intermediate phase (Fig. 7e). This approach facilitates large separations through stress concentration in the hard phase, leveraging the high ionic charge mobility in the soft and intermediate phases. Remarkably, the ionogel elastomer achieved an extraordinary piezoelectric efficiency of about 6.0 mV kPa$^{-1}$ and a power density of 1.3 $\mu$W cm$^{-3}$. The ionogels can also serve as energy storage and buffering components in piezoelectric generators, further optimizing the operating efficiency of the entire power-generation system and offering numerous innovations and opportunities in the field of piezoelectric power generation.

#### Thermoelectric devices

Thermoelectric devices exploit a physical phenomenon and associated technology that converts thermal energy into electrical energy, or vice versa, through the movement of carriers within a specific material (thermoelectric material), and they have significant applications in energy-harvesting devices [131–133]. The application of ionogels in thermoelectricity holds considerable promise across various dimensions. Ionogels exhibit impressive Seebeck coefficients, enabling them to generate substantial thermoelectric potentials due to the differential thermal mobility of anions and cations. Their inherently low thermal conductivity is advantageous, as it helps maintain the temperature gradient, thereby enhancing the thermoelectric conversion efficiency [134–138]. Furthermore, ionogels demonstrate excellent flexibility and processability, enabling their integration with flexible substrates to create wearable thermoelectric devices that can conform to the human body or irregular surfaces. Such devices are capable of effectively harvesting low-grade heat energy and converting it into electricity [139]. Additionally, ionogels can function as self-powered units within distributed energy systems, thereby improving the efficiency of charge and heat transfer [140–142]. Consequently, these attributes collectively contribute to comprehensively advancing the field of thermoelectricity. Yan *et al.* [143] designed a thermoelectric ionogel with a high Seebeck coefficient (32.4 mV K$^{-1}$) based on 1-ethyl-3-methylimidazolium dicyandiamide and the ion redox pair [Fe(CN)$_{6}]^{4-}$/[Fe(CH)$_{6}]^{3-}$. This innovative ionogel was integrated with passive radiative-cooling technology to create a thermal battery capable of continuous electricity generation. It can continuously generate electricity under varying weather conditions across a wide temperature range ($-40\, ^\circ$C to $90\, ^\circ$C), achieving a normal power density of 25.84 mW m$^{-2}$K$^{-2}$ (Fig. 7f). Ionogels exhibit significant potential in energy-harvesting devices. Future research directions include the exploration of new high-performance ionogel materials, optimization of device structures and fabrication processes, multifunctional integration and the development of environmentally friendly materials [58,144]. These endeavors are expected to facilitate the realization of self-powered smart systems, addressing the needs of applications such as wearable devices and biomedicine.

### Energy-storage devices

Ionogels are pivotal in energy-storage technology due to their unique physicochemical properties, which are essential for applications in batteries, supercapacitors and other energy-storage systems. As electrolytes, ionogels offer high ionic conductivity, facilitating the rapid and efficient ion transfer between electrodes. Their excellent flexibility and processability enable them to conform to diverse electrode shapes and sizes, making them particularly advantageous for flexible energy-storage solutions. Additionally, ionogels demonstrate remarkable chemical and thermal stability, allowing them to maintain performance over extended periods and mitigating electrolyte degradation and performance decline. Moreover, by integrating ionogels with other functional materials, it is possible to boost energy and power densities. This capability not only optimizes existing energy-storage technologies but also provides innovative avenues and robust support for their continued development.

#### Capacitors

**Figure 8.**
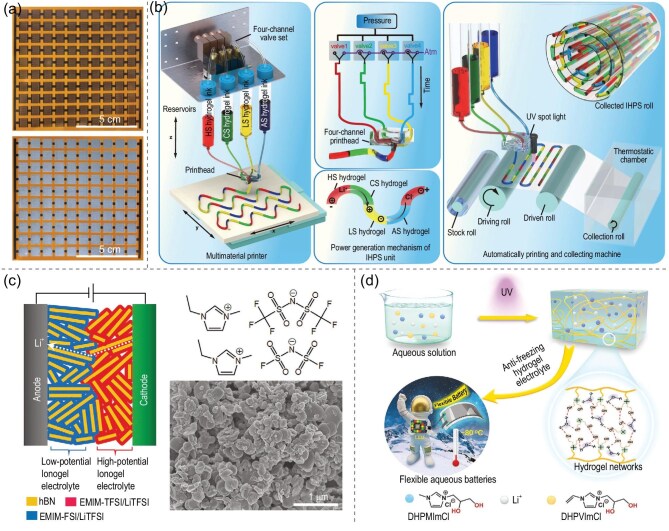
(a) Top: digital photographs of a 10S $\times$ 10P array of screen-printed graphene interdigitated electrodes before the high-temperature hexagonal boron nitride (hBN) ionogel was printed; bottom: digital photographs of a fully printed 10S $\times$ 10P micro-supercapacitor array, with the hBN ionogel printed on top of the graphene interdigitated electrodes [62]. Copyright 2023, Wiley-VCH GmbH. (b) Schematics of the consecutive multi-material printing setup, showing the programmable-controlled four-channel valve set for dynamic switching between different inks, the power-generation mechanism of the consecutively printed ionic hydrogel power source (IHPS) unit and the automated multi-material printing system with collection modules [160]. Copyright 2024, Springer Nature. (c) Layered heterostructure ionogel electrolyte for solid-state lithium-ion batteries [18]. Copyright 2021, Wiley-VCH GmbH. (d) Preparation and components of the anti-freezing hydroxyl-functionalized poly(ionic liquid) hydrogel. Using the PIL-OH hydrogel, the flexible aqueous lithium-ion cell exhibited outstanding low-temperature performance and could operate in environments as low as $-80\, ^\circ$C [64]. Copyright 2022 Wiley-VCH GmbH.

Ionogels can be employed as electrolytes for high-performance capacitors, in which their high ionic conductivity allows for the rapid migration of ions through the ionogel network, thereby increasing the power density [145–147]. Meanwhile, a judiciously selected ionic liquid can expand the electrochemical window, allowing stable operation at high voltages and thus increasing the energy density [148,149]. Furthermore, their flexible network endows the capacitor with excellent mechanical deformation capability, making it adaptable to operations such as bending and folding. Hersam *et al.* [62] demonstrated a micro-supercapacitor that can maintain stable mechanical flexibility at $180\, ^\circ$C by continuous high-speed screen printing of conductive graphene electrodes and a high-temperature hexagonal boron nitride (hBN) ionogel electrolyte. The specific surface capacitance almost reached 1 mF cm$^{-2}$ (Fig. 8a). Studies have shown that certain ionogel-based supercapacitors exhibit remarkable mechanical flexibility and capacity, with minimal performance degradation even after tens of thousands of charge/discharge cycles [150]. This durability and energy-storage efficiency underscore their potential as an ideal choice in the field of flexible electronics.

#### Flexible batteries

Ionogel serves as an electrolyte material that significantly boosts the performance of flexible batteries due to its high ionic conductivity and excellent mechanical properties. Flexibility, safety, stability and energy-conversion efficiency play crucial roles in advancing the development of flexible batteries, which is essential for their practical application and widespread adoption. Its high ionic conductivity facilitates the rapid and efficient ionic transference between the electrodes, which is vital for improving charging and discharging performance [151–153]. Additionally, the ionogel ensures excellent contact with electrode materials, thereby reducing interfacial resistance and optimizing both the overall battery performance and cycling stability. The unique structure and properties of ionogels, when used as electrolytes in metal batteries, significantly inhibit lithium dendrite formation and growth, thereby minimizing the risk of short circuits [154–157]. Furthermore, its high mechanical strength enables it to withstand the stress of charging and discharging, stabilizing the electrode structure. Ionogels also exhibit excellent thermal stability and a broad operating temperature range, maintaining high ionic conductivity even under harsh conditions. This reliability ensures that the battery functions reliably across a range of environmental conditions [158,159]. Meanwhile, by selecting appropriate ionic liquids, a wide electrochemical window for batteries can be achieved, enabling safe and stable operation under high-voltage and high-energy-density conditions, as well as continuous multi-material printing (Fig. 8b) [160].

Hersam *et al.* [18] designed an ionogel electrolyte featuring a layered heterogeneous structure composed of imidazolium-based ionic liquids to broaden the electrochemical stability window of all-solid-state lithium-ion batteries, thereby increasing their operating voltages (Fig. 8c). The structure of ionogels also provides excellent mechanical flexibility, enabling the batteries to withstand bending and folding without damage, thus making them suitable for flexible electronics. This improves their safety, substantially reducing the risks posed by traditional liquid electrolytes due to their non-volatile and non-leakage properties. As shown in Fig. 8d, Yan *et al.* [64] developed an ionogel based on hydroxyl functionality (PIL-OH) with an ultra-low-temperature-tolerant electrolyte, and the capacity retention rate of the prepared aqueous lithium-ion battery reached 93% after 200 cycles at $-80\, ^\circ$C. Collectively, these advantages have driven the development of ionogel-based flexible lithium-ion batteries, offering new solutions for wearable devices and flexible robots.

To further optimize the performance of ionogels in energy-storage devices, researchers are actively exploring various innovative structural design strategies. One approach involves constructing 3D porous structures to significantly increase the specific surface area and connectivity, thereby dramatically enhancing the ionic conductivity and electrochemical performance [59]. Another strategy is to develop composite electrolytes by combining ionogels with other functional materials (e.g. conductive polymers and 2D materials) to achieve multifunctional integration and improve the overall performance [161,162]. Additionally, the use of micro- and nanostructure processing techniques not only helps to reduce the device size but also effectively improves the ionic conductivity and power density. Finally, the preparation of solid-state ionogel electrolytes through solid-state processing, with no leakage or volatilization, further ensures battery safety [74,143]. It is expected that ionogels will demonstrate greater application potential in the fields of flexible batteries and supercapacitors and drive the development of flexible-electronics technology. Researchers will continue to explore novel ionic liquids, ionogel networks and their composite structures for the comprehensive enhancement of the performance of energy-storage devices to meet the growing demand for flexible electronics.

### Smart devices

With their unique stimulus-responsive properties, ionogels exhibit broad application prospects in the field of intelligent systems. They can respond to external stimuli, including temperature, pH, light, electric and magnetic fields, by altering their physical or chemical properties (for instance, shape, color, electrical conductivity and mechanical properties), thereby realizing intelligent functions.

#### Actuators

**Figure 9.**
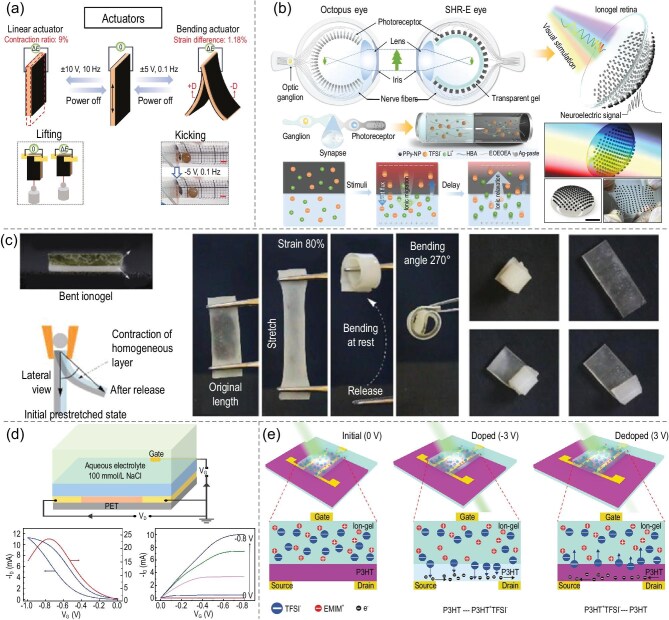
(a) The trilayer ionic electroactive polymer device interpenetrating liquid-crystal elastomers (i-EAD-IPN-LCE) as an electroactive actuator and bending sensor [39]. Copyright 2024, Wiley-VCH GmbH. (b) Bioinspired self-powered hemispherical retinomorphic eye (SHR-E) structure with an ionogel heterojunction retina [167]. Copyright 2024, Springer Nature. (c) Programmable actuating devices based on the shape-memory behavior of PAA ionogels [172]. Copyright 2025, Chinese Chemical Society. (d) Schematic diagram of an organic electrochemical transistor (OECT) with ionogel, the output curves of OECT with PH/3LiT ionogel as the interfacial layer and the transconductance (gm) curves [73]. Copyright 2023, Wiley-VCH GmbH. (e) Schematic of the modulation mechanism of the device under different gate voltage (Vgs) amplitudes [183]. Copyright 2023, American Chemical Society.

Ionogel-based actuators, capable of converting external stimuli into mechanical movements, have a broad range of potential applications in areas such as soft robots, artificial muscles, microfluidics and biomedical devices. Their working mechanism relies on the electrochemical actuation or environment-responsive properties of ionogels [163–166]. Li *et al.* [39] successfully developed tunable, electrically bifunctional actuators and sensors by preparing an interpenetrating network of liquid-crystal elastomers and ionogels, integrated with a three-layer ion-conducting polymer smart device (i-EAD) (Fig. 9a). As show in Fig. 9b, Huang *et al.* [167] prepared a self-driven hemispherical retinal-morphology eye with ionogel heterojunctions as photoreceptors by combining neuro morphological principles with retina engineering and ionic elastomers. In an electrochemically driven actuator, an external electric field can induce ion migration within the ionogel network, thus resulting in a volumetric or shape change [168–170]. In environmentally responsive actuators, external stimuli (e.g. temperature, pH and light) contribute to the dissolution or contraction of the ionogel, thereby generating mechanical motions [171].

Multiphase ionogels, distinguished by their high strength and broad range of switchable stiffness, can be synthesized by adjusting parameters within the phase diagram of a liquid-liquid phase-separation ionogel system. These ionogels can be engineered into multi-phase composites with bilayer or sandwich structures, which are particularly useful in the development of smart devices. For instance, heterogeneous ionogels containing specific microstructures and micro-dopants can exhibit shape and stiffness memory [37]. The ability to alter stiffness impacts the sensor’s pressure range, detection limit and resolution under compressive stress. Additionally, ionogels can be enhanced with antifreeze properties and electrical conductivity, making them suitable for applications in various fields, including soft robotics, smart devices and artificial muscles. The integration of these properties highlights the broad application potential of ionogels in advanced technological domains. As shown in Fig. 9c, Liu *et al.* [172] proposed a strategy to manipulate the metastable ionogels by positioning them at the Berghmans point in the phase diagram through the construction of a liquid-liquid phase-separation system interacting with the polymer glass transition. By creating multiphase composite ionogels with a bilayer or sandwich structure, smart devices with programmable actuation behaviors were constructed, achieving operating densities as high as 161.5 kJ m$^{-3}$, which are superior to those of conventional gel actuators or even animal muscle tissues. These nonvolatile ionogels, with controllable metastability and excellent mechanical strength, have potential applications in fields such as soft robotics, smart devices and artificial muscles.

#### Ionic-gated transistor

When an electric field is applied across the ionogel, the redistribution of anions and cations occurs at the electrode-electrolyte interface. This redistribution leads to the aggregation of oppositely charged ions near the electrode surface, resulting in the formation of an extremely thin double electric layer structure [173,174]. From a physicochemical perspective, the thickness of this bilayer is typically in the nanometer range, which is significantly smaller than that of conventional inorganic dielectric materials [175,176]. Consequently, the capacitance value is dramatically increased. This enhanced capacitance allows for effective regulation of channel carriers at lower voltages, thereby reducing the transistor threshold voltage and operating voltage, minimizing power consumption and expanding potential application scenarios [177,178]. As shown in Fig. 9d, Chi *et al.* [73] prepared ionogel films as interfacial layers in organic electrochemical transistors (OECTs), consisting of a gel matrix of poly(vinylidene fluoride)-co-hexafluoropropylene and two ionic liquids (lithium bis(trifluoromethylsulfonyl)imide and 1-ethyl-3-methylimidazolium bis(trifluoromethylsulfonyl)imide). The two ionic liquids share the same hydrophobic anion but have different counterbalancing cations. Mechanistic studies have shown that high anion content and small cation size in ionogels favor the electrochemical doping of p-type OECTs. Therefore, hydrophobic conjugated polymers can be directly applied to OECTs while maintaining their hole mobility, without further grafting steps, by taking advantage of ionogels. In addition, OECT performance can be easily tuned by varying the types and ratios of the gel interfacial layer. Furthermore, the ions in the ionogel can migrate under an external electric field, thereby impacting the concentration and mobility of transistor channel carriers. By strategically designing the ionic species, concentration and mobility within the ionogels, it is possible to accurately regulate key electrical properties such as threshold voltage, subthreshold swing and switching speed of transistors [179]. Jeong *et al.* [180] presented a fully parallel-processed synaptic array with reduced control complexity for coordinated motion. The synaptic array was prepared by attaching eight ionogel-based synaptic transistors to an ionogel medium. By regulating the ionic motion features, parallel processing of signals and multi-driver control can be achieved. This approach offers a novel pathway for designing high-performance transistors.

Furthermore, the unique structure of ionogels imparts them with exceptional flexibility, stretchability and stress-buffering capabilities, making them highly suitable for application in flexible-electronics devices. In these devices, ionogels can adapt and maintain stable dielectric properties even when the transistor undergoes multiple deformations. This adaptability ensures the normal functioning of transistors in complex deformation environments, such as those found in wearable devices and foldable displays. When exposed to external mechanical stress, the polymer network within the ionogel effectively disperses the stress, thereby preventing microcracks or delamination [181,182]. Yang *et al.* [183] reported a flexible organic electrochemical synaptic transistor array with optical signaling as a physical expression of bionic synapses, used for neuromorphic optical image processing (Fig. 9e). The authors demonstrated the fabrication of optically readable organic electrochemical synaptic transistor (OR-OESTs) arrays with planar side-gate structures on flexible polyethylene terephthalate substrates using screen-printed ionogel as the gate medium. De-noising of optical images, as well as contrast enhancement, was realized in OR-OEST arrays. This ionogel protective mechanism safeguards the structural integrity of the device, ensures good contact between materials and maintains stable electrical properties. Consequently, the device service life is extended, providing robust support for the stable operation of flexible-electronics devices.

#### Luminous and color-changing devices

**Figure 10.**
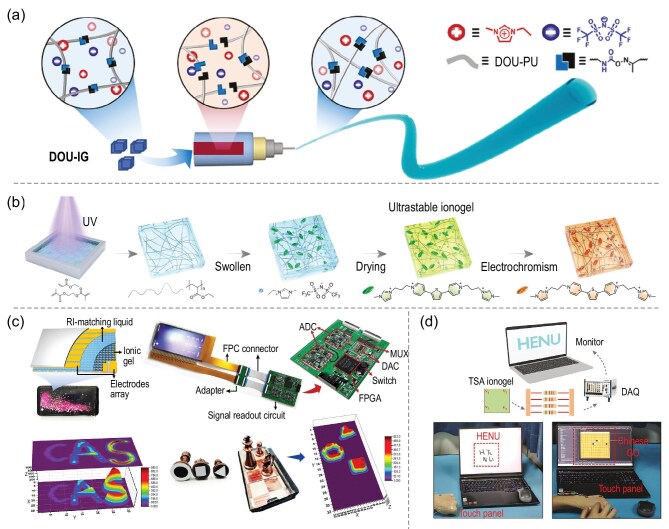
(a) Illustration of the melt spinning of dimethylglyoximeurethan ionogel (DOU-IG) fibers [47]. Copyright 2023, Wiley-VCH GmbH. (b) Schematic illustration of the fabrication process for an ionogel for a visible strain sensor, integrating electrochromism, electrofluorochromism and strain sensing [188]. Copyright 2022, Chinese Chemical Society. (c) Schematic diagram of a touch-screen structure, circuit design of the touch system and pressure thermogram of touch capture [196]. Copyright 2022, Springer Nature. (d) Schematic diagram of a self-powered epidermal touch panel composed of an ionogel film, resistances, data acquisition system (DAQ) and monitor, along with demonstrations of the multifunctional touch panel in different scenarios [197]. Copyright 2023 Wiley-VCH GmbH.

Ionogels can be integrated with light-emitting or color-changing materials to obtain flexible devices with luminescent or color-changing functions [184,185]. For example, by weaving ionogel-based electroluminescent devices into garments, light-emitting clothes can be prepared for nighttime safety warnings or fashion decoration [94,186]. You *et al.* [47] prepared adapted covalently crosslinked ionogel fibers based on dimethylglyoximeurethane (DOU) groups (DOU-IG fiber). Multifunctional flexible smart electronics, such as sensors, friction nanogenerators and electroluminescent displays, were integrated using prepared ionogel fibers and used for motion monitoring, energy harvesting and human-computer interaction (Fig. 10a). In addition, ionogel-based color-changing devices capable of changing colors according to ambient temperature, light or electrical signals are widely used in wearables, smart homes, automotive interiors and dynamic displays for real-time color change and functional enhancement [187]. For example, He *et al.* [188] integrated a thiophene violet-containing elemental violet essence electrochromic material with an ionogel based on violet essences containing sulfur group elements, yielding a violet-essence-based ionogel with strain-sensing properties capable of color change (Fig. 10b). The versatility and superior performance of ionogel in electrochromic devices not only offer new ideas for the innovation of modern smart materials but also pave the way for future sustainable development and the realization of high-performance electronic devices.

#### Tactile feedback devices

Because of their excellent electrical conductivity, mechanical flexibility and biocompatibility, ionogels exhibit significant potential in intelligent haptic-feedback systems [189–191]. By simulating human haptics through electrical or mechanical stimulation, these systems provide an authentic interaction experience for applications in virtual reality, augmented reality, robotics and human-computer interaction [192–195]. By employing ionic liquids with high ionic conductivity and good biocompatibility, along with adaptable ionogel networks, the responsiveness and sensitivity of the system can be improved. Pan *et al.* [196] developed a flexible ultra-transparent tactile-sensor device based on the principle of iontophoresis, utilising a surface-structure design and refractive-index-matching scheme. Ultrahigh optical transparency (96.9%) and a device sensitivity of up to 83.9 kPa$^{-1}$ were achieved (Fig. 10c). In practical applications, the ionogel-based tactile-feedback system can enhance the immersion of virtual reality and augmented reality by simulating the tactile sensation of virtual objects. As shown in Fig. 10d, Pan *et al.* [197] developed a transparent, self-healing, frost-resistant ionogel composed of a fluorine-rich ionic liquid and a fluorocarbon elastomer. The ionogel was seamlessly transformed into an autonomous multifunctional epidermal touch panel using Faraday’s law of induction and the inherent antennae properties of the human body. This touch panel provides input functionality through text input and participation in the Chinese Go game. Meanwhile, the system can be employed for haptic perception in robotics, enhancing the naturalness of human-robot interaction. In addition, it enables the development of novel human-computer interaction interfaces, such as haptic displays and keyboards, to promote the user experience.

## CONCLUSION AND PERSPECTIVE

This review provides a comprehensive overview of the research advancements in ionogels for flexible device applications. The efficacy of these flexible devices is intrinsically linked to the properties of ionogels, necessitating a detailed analysis of their composition and structure. We comprehensively summarized the structure-property-application relationships of ionogels, which comprise various ionic liquids interacting with diverse network architectures. The designability of ionogel components and structures facilitates multi-scale property modulation through structural modification. This adaptability results in outstanding mechanical properties, stability, electrochemical performance and responsiveness to stimuli. Consequently, ionogels can meet the diverse requirements of flexible devices and establish a foundation for their extensive application in complex scenarios.

Despite the significant advancements in the development of ionogels for flexible devices, several challenges persist. A principal concern is the insufficient synergistic integration of multiple functions, which gives rise to signal interference and difficulties in attaining a balance in energy conversion efficiency, thereby rendering the achievement of a high level of functional integration in practical applications. Additionally, the long-term durability of ionogels demands improvement, as their performance tends to deteriorate over time due to factors such as mechanical stress, temperature fluctuations and exposure to corrosive chemical environments. These conditions result in diminished electrical conductivity and weakened mechanical strength, making it challenging to maintain long-term stability and peak performance. Furthermore, the self-healing capabilities of ionogels are currently suboptimal, as the healing process is sluggish and the restoration of performance is partial, failing to meet the requirements of wearable devices and other applications frequently subjected to external forces. Meanwhile, the environmental friendliness of ionogels requires improvement. Some materials used in ionogels exhibit limited degradability, and the manufacturing procedures may negatively impact the environment, falling short of the standards for comprehensive environmental sustainability. The biocompatibility and toxicity of ionogels primarily arise from intrinsic toxicity, ionic-liquid leakage, inflammatory/foreign body reactions, individual variability and uncertainties regarding long-term toxicity. These challenges can be addressed by selecting low-toxicity ionic liquids (choline-based, amino-acid-based), using biocompatible polymers (e.g. amphoteric ions, gelatin, PEG), adjusting mechanical properties, developing low-toxicity ionic liquids, optimizing network leak prevention and developing degradable systems. Such enhancements c could meet the demands of applications such as flexible-electronics skin.

The future application of ionogels in flexible devices holds significant promise. A thorough investigation into the intrinsic mechanism of ionogels could facilitate the design of ionogels with tailored properties. To systematically characterize the ionic conduction pathways, interactions between the polymer network and ions, and energy dissipation mechanisms, advanced techniques such as *in situ* characterization, high-resolution spectroscopy and multi-scale simulation are urgently required. These approaches would provide a solid theoretical foundation and data guidance for optimizing performance, enhancing stability and innovatively designing ionogel-based flexible-electronics devices. To achieve practical multifunctional integration, it is essential to combine innovative material design and device architecture, enabled by nanotechnology and composite methods with intelligent control systems. This approach is expected to meet the diverse requirements of various applications. Ensuring long-term stability requires an in-depth study of aging mechanisms. By introducing new stabilizers or modified materials and employing monitoring technology to track performance changes in real time, ionogels can maintain stable operation in complex environments. Enhancing self-healing capabilities involves exploring new mechanisms and material systems, potentially drawing inspiration from bionics, to achieve rapid and complete healing and performance recovery. This advancement would significantly improve the service life and reliability of devices. Furthermore, the development of green chemistry offers the potential to create environmentally friendly and degradable materials, ensuring sustainability throughout the entire lifecycle, from production to disposal. This approach promotes the broader application of ionogels across various fields.

## Supplementary Material

nwaf541_Supplemental_File
