# Supplementary material for: Ionogel-based flexible electronics
Source: Natl Sci Rev. 2025 Dec 3;13(4):nwaf541. doi: 10.1093/nsr/nwaf541 (PMC12875126; doi:10.1093/nsr/nwaf541)
Supplement: nwaf541_Supplemental_File [file nwaf541_supplemental_file.pdf]

Supplementary Information

Ionogel-based Flexible Electronics

Qinbo Liu[1], Xu Ou[1], Yingjie Zhou[1,*] and Feng Yan[1,2,*]

[1]State Key Laboratory of Advanced Fiber Materials, College of Materials Science and Engineering, Donghua University, Shanghai 201620, China

[2]Jiangsu Engineering Laboratory of Novel Functional Polymeric Materials, College of Chemistry, Chemical Engineering and Materials Science, Soochow University, Suzhou 215123, China

*Corresponding author: F. Yan, E-mail: fyan@suda.edu.cn.

Inventory of Supplementary Information:

Supplementary Figure S1. Schematic illustration of the application of ionogels in flexible electronics.

Supplementary Figures

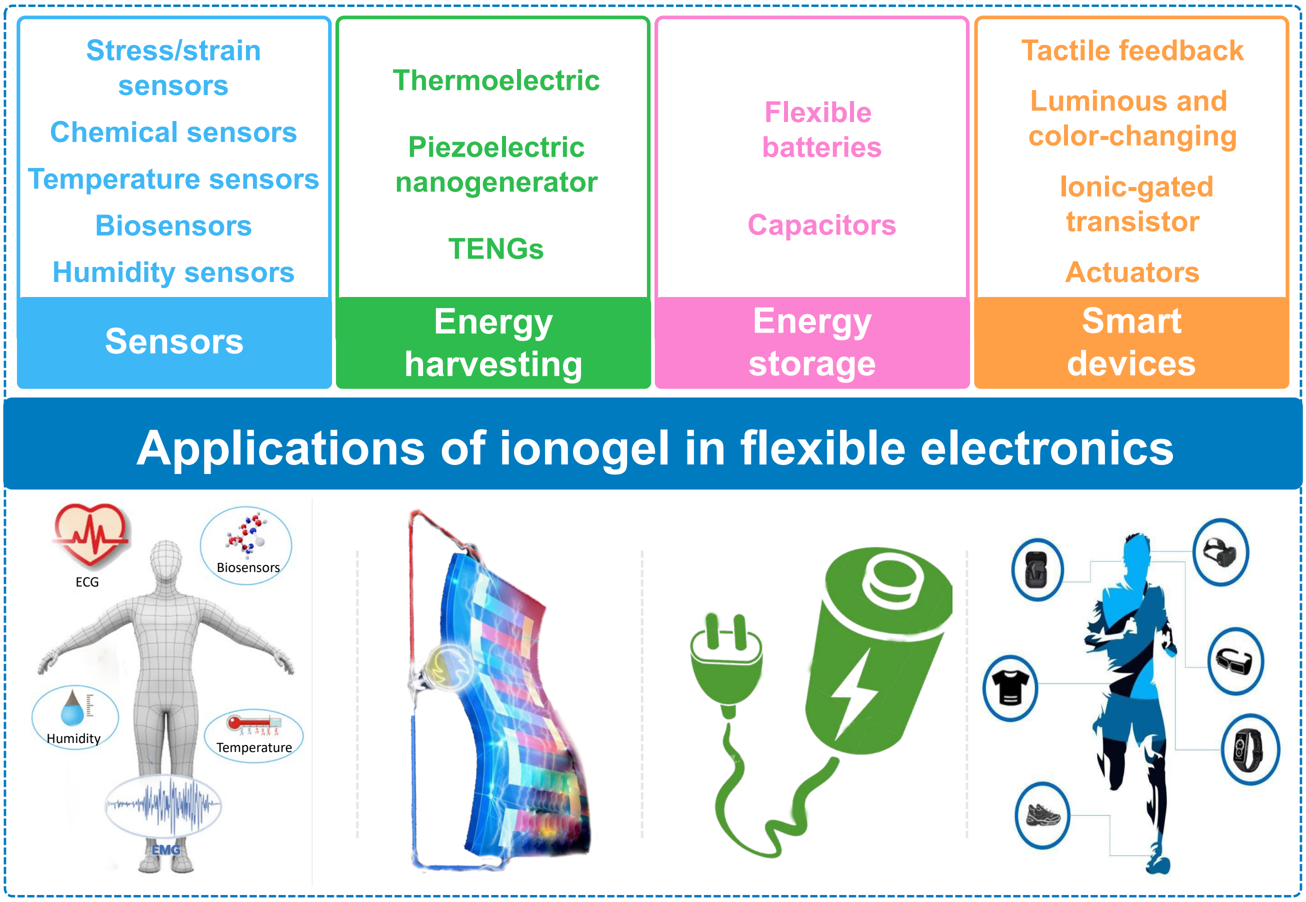

Figure S1. Schematic illustration of the application of ionogels in flexible electronics.
